# Association between personality traits, dental caries, and oral health-related behaviors among underserved Egyptian adults: a cross-sectional study

**DOI:** 10.1186/s12903-026-09790-6

**Published:** 2026-09-26

**Authors:** Amira H Elwan, Rana Mahrous, Fadia Alawad, Aml Hatem, Rowan Elshahawy, Wafaa Essameldin Abdelaziz, Maha El Tantawi

**Affiliations:** Department of Pediatric Dentistry and Dental Public Health, Faculty of Dentistry, Alexandria University, Champolion St., Azarita, Alexandria, 21527 Egypt

**Keywords:** Dental caries, Personality traits, Big Five, Toothbrushing, Dental visits, Sugar consumption, Extraversion, Agreeableness, Openness, Neuroticism, Conscientiousness

## Abstract

**Background:**

The Big Five personality traits comprise five broad dimensions of personality namely openness, conscientiousness, extraversion, agreeableness, and neuroticism which may influence oral health. Examining these influences in underserved communities helps inform the design of tailored oral disease prevention strategies. This study assessed the association between personality traits on one hand and dental caries as well as oral health behaviors on the other hand, among underserved Egyptian adults.

**Materials and methods:**

A cross-sectional study was conducted in three governorates (Alexandria, Elbeheira and Matrouh) in Northwestern Egypt between November and December 2025. Data were collected, in community settings, from mentally healthy, adults, using a self-administered questionnaire assessing personality traits by the Big Five Inventory–2 Extra-short Form, as well as the Arabic translated WHO adult form for oral health behaviors (toothbrushing, dental visits, and sugar consumption). Dental caries experience was assessed using the DMFT index. The independent variables were personality traits, while the dependent variables were caries and the three oral health behaviors in four separate models. Potential confounders (age, gender, educational level, and residence) were controlled. Four regression models (linear and binary logistic) were fitted and regression coefficients (B), Adjusted odds ratio (AOR), 95% confidence intervals (CI), model fit statistics (R²/pseudo-R², as appropriate to model type)were calculated.

**Results:**

A total of 820 adults were included (mean age = 36.5 ± 13.0 years), of whom 70.2% were females, 59.1% brushed once or more daily, 60.6% had visited a dentist in the previous year, the mean sugar consumption score was 3.85 ± 2.06 and the mean DMFT index was 5.16 ± 5.04. Agreeableness showed the highest mean score (11.53 ± 1.97), while openness had the lowest mean score (9.64 ± 2.27). Neuroticism was significantly associated with less toothbrushing (AOR = 0.90, *p* = 0.003). Openness was significantly associated with higher dental visits (AOR = 1.08, *p* = 0.03) and sugar consumption (B = 0.12, *p* < 0.001). Conscientiousness was significantly linked to reduced sugar intakes (B=-0.15, *p* < 0.001).

**Conclusion:**

Personality traits were significantly associated with the caries experience and oral health behaviors of underserved Egyptian adults, which may help targeting individuals for preventive interventions.

## Introduction

Dental caries is the most common noncommunicable disease (NCD) worldwide, affecting 2.5 billion people [1]. Oral health-related behaviors, mainly tooth brushing, sugar ingestion, frequency of dental appointment, affect the occurrence of oral diseases. Moreover, literacy levels, cultural and environmental factors are social determinants of health engaged also with oral health [2–4]. Beyond traditional behavioral and environmental determinants, psychological and personality characteristics are increasingly recognized as important influences on oral-health outcomes. Literature identified Big five personality characteristics as: conscientious (individuals who tend to be diligent and are usually task and goal-directed), neurotic (individuals who tend to feel depressed, anxious, impulsive, and may display hostility), open (individuals who are imaginative and are open for new ideas), extroverted (individuals being sociable and outgoing), and agreeable (individuals who tend to be altruistic and warm) [5].Personality traits exhibit stability across adulthood and remain relatively consistent over time and can influence long-term behaviors in adult populations [4]. This study is grounded in the Five-Factor Model of personality, which proposes that stable dispositional traits shape health-relevant cognition, self-regulation, and stress reactivity, and thereby influence engagement with preventive health behaviors over the life course [6].

Personality traits can shape oral health through a combination of behavioral, emotional, and coping mechanisms. Extroverted people are more likely to engage in preventive health practices [7]. Highly agreeable individuals tend to adhere more consistently to health instructions [8]. Highly open individuals can explore preventive strategies [9]. Conscientious adults demonstrate more consistent engagement in positive health behaviors [10]. In contrast, adults with high neuroticism tend to engage in unhealthy behaviors and experience negative health consequences, including difficulties in making informed decisions [11].

Greater extraversion was significantly associated with increased dental caries among adolescents in Saudi Arabia [12] and was also significantly associated with improvement in Austrian oral lichen planus patients along the course of treatment [13]. Higher extraversion was also significantly associated with regular dental visit attendance among German elderly [14].

Meanwhile, neuroticism was associated with higher number of decayed and missing teeth among adult Mexicans [4]. An Indian study showed neurotic patients with periodontal diseases had non-significantly more gingivitis and plaque accumulation than extroverted or open patients. A study showed that individuals with higher neuroticism tended to use more healthcare services in a sample of American adults [15]. On the other hand, Spanish adults who brushed their teeth more than once per day were more agreeable than those who only brushed once a day [16].

Personality traits also relate to dietary habits: A meta-analysis indicated that higher neuroticism was associated with less healthy dietary patterns and emotional eating, while extraversion, openness, agreeableness, and conscientiousness relate to healthier eating behaviors, including lower sugar consumption [17].

Egypt reported, in a national survey, a mean DMFT of 5.5 in permanent dentition on adults, with 78.2% having DMFT > 0 and 39% presenting with at least one untreated carious tooth [18]. Underserved populations are communities that receive less than adequate healthcare services due to social, economic, cultural and/or linguistic barriers to accessing healthcare services, lack of familiarity with the healthcare delivery system, living in locations where providers are not readily available or physically accessible [19]. In 2022, multidimensional poverty affected 21% of the Egyptian population, with 19% deprived of basic services [20]. Evidence on the relationship between psychosocial influences and oral health in Egypt is limited [21]. No previous studies have assessed the relationship between personality traits, dental caries experience, and oral health–related behaviors among underserved Egyptian adult populations. Understanding how personality traits are associated with oral health behaviors may help development of tailored, person-centered oral health services that complement service provision and enhance the effectiveness of preventive interventions in underserved settings.

The novelty of this study lies not in demonstrating that individual personality–behavior associations exist, which is already established in the literature reviewed above, but in the simultaneous assessment of caries experience and three distinct oral health behaviors against all five personality traits within a community-recruited, underserved adult population.

The aim of the current study was to assess the association between personality traits on one hand and dental caries experience and oral health-related behavior on the other hand among underserved Egyptian adults. Given the cross-sectional nature of the study, the observed associations should not be interpreted as causal relationships. The null hypothesis was that there would be no association between personality traits, dental caries experience and oral health-related behavior among underserved Egyptian adults.

## Materials and methods

### Study design and ethical considerations

A cross-sectional study was conducted in Northern Egypt: Alexandria (coastal, mainly urban, population size = 5,441,385), Elbeheira (Nile Delta, predominantly rural, population size = 6,927,000), and Matrouh (Western Desert, urban with Bedouin communities, population size = 530,270) [22]. Data were collected between November and December 2025. Ethical approval was obtained from the Research Ethics Committee at the Faculty of Dentistry, Alexandria University, Egypt (#1196-11/2025). Permissions from the local authorities were secured. The study was conducted in full accordance with the Helsinki declaration. A consent form was distributed to study participants. Objectives, benefits, and risks of the study were explained in detail. All participants were assured of data confidentiality and informed of their right to withdraw at any time. The study is reported according to the Strengthening the Reporting of Observational Studies in Epidemiology (STROBE) guidelines for observational studies [23].

### Participants

Mentally healthy and literate adults aged above 18 years were included. Individuals with orthodontic appliances were excluded due to the difficulty of examining all teeth surfaces. Participants were recruited through youth centers, public service facilities, and community outreach programs in Alexandria, El Beheira, and Matrouh Governorates, where they received dental services because they had limited access to routine dental care. During data collection, local guides assisted with the recruitment of participants residing in the area

Study Variables

### Study variables

#### Outcome variables

There were four outcome variables: caries experience, frequency of toothbrushing using fluoridated toothpaste, dental visits during the last year and the score of sugar consumption. Dental experience in permanent teeth of this adult population was assessed using the DMFT index, according to the WHO criteria [1]. Three examiners (A.H.E., R.M., and F.A) were calibrated with a gold standard examiner, then the inter-examiner reliability was assessed (Kappa score = 0.92), and intra-examiner reliability was tested after 2 weeks (Mean Kappa score = 0.90), indicating excellent agreement among and within examiners across time [24].

Oral health behaviors were assessed using the Arabic validated version of the WHO questionnaire adult form [25], including frequent toothbrushing using fluoridated toothpaste (classified into at least once daily or less), dental visits during the last year (yes or no), and sugar consumption (at least once daily consumption of 8 types of sugary foods/snacks: biscuits or cakes, fruits, jam or honey, carbonated beverages or lemon juice, candy, sugar-sweetened milk and sugar-sweetened hot beverages, sugar-added chewing gums). The sugar consumption score was created by summing the number of products that were consumed at least once daily. The scores ranged from 0 (lowest) to 8 (highest) sugar consumption.

#### Explanatory variable

The five personality traits were assessed using the Big Five Inventory–2 Extra-short Form (BFI-2- XS) [26]. The scale asks participants to describe themselves on 15 items using a five-point Likert scale ranging from 1 = strongly disagree to 5 = strongly agree. Each trait is covered by 3 items, including extraversion (quietness, leadership, and energy), agreeableness (compassion, rudeness, and good faith in people), conscientiousness (disorganization, procrastination, and reliability), neuroticism (stress, depression, and emotional stability), and openness (artistic inclination, abstract thinking, and creativity). Five Items are reverse-scored. The total score of each trait is calculated by summing the scores of its items, where 3 = weakest and 15 = Strongest presence of the personality trait. The questionnaire was translated into Arabic by a bilingual translator and subsequently back-translated into English by a different independent bilingual translator to assess linguistic equivalence between the two versions [27]. The Arabic version was then pilot tested before its use in the main study to assess its clarity and comprehensibility. The internal consistency of the Arabic version was assessed in the pilot sample, with Cronbach’s α value = 0.85, indicating good internal consistency [28].

#### Potential confounders

Background information was collected, including gender (male or female), age in years at last birthdate, educational level (Below university or university and above), and residence (rural or urban). The full questionnaire was pilot tested on 30 individuals to assess the comprehensibility of the questionnaire and study feasibility. Their data were excluded from the final analysis.

#### Field procedures and quality control

The questionnaires were anonymous, paper-based and self-administered. Participants were informed were provided with general instructions for its completion. Questionnaire administration was supervised on-site by trained data collectors, who were available to clarify the wording of items without prompting or influencing participants’ responses, and also checked the responses for completeness before submission. Participants were asked to complete any missing items where feasible.

Clinical examinations were performed by three calibrated examiners under daylight using WHO dental probes and appropriate infection-control procedures, including sterilization of instruments and the use of disposable examination gloves. After each participant completed the questionnaire and clinical examination, All participants received toothpaste and toothbrushes with health education instructions and were referred for treatment if needed. Data from the questionnaires and clinical examinations were entered into an online platform, KoboToolbox, and subsequently downloaded for statistical analysis.

#### Sample size estimation

Sample size was calculated assuming an 80% power and 95% confidence interval (CI) to assess the association between the five major personality traits and dental caries. It was assumed that the effect size is small (Cohen’s f² = 0.02) [29] and up to 9 explanatory variables will be used to assess the association. Using the G power 3.0.10. calculator for multiple linear regression, the sample size was estimated to be 791, increased to 870 individuals to compensate for 10% incomplete responses [30, 31].

#### Statistical analysis

Normality of the quantitative variables was tested. There were variables with normal distribution, including age, sugar consumption score, and personality trait scores, and variables with non-normal distribution, including the DMFT scores. To assess the association with the binary oral health behaviors, frequent toothbrushing and dental visits last year, the independent *t*-test and the Mann–Whitney *U* test were used with the normally and non-normally distributed variables respectively. The associations of the binary outcome variables with the categorical variables, gender, educational level, residence, were assessed using the Chi-square test. Correlations between continuous variables were evaluated using Pearson correlation for sugar consumption score and Spearman correlation for caries experience (DMFT).

Four regression models were created. In model 1(negative binomial regression), the outcome variable was caries experience (DMFT). In models 2 and 3 (binary logistic regression), the outcome variables were frequent toothbrushing and dental visit last year, respectively. In model 4 (linear regression, the outcome variable was the sugar consumption score. In models 1 and 4, regression coefficients (B), and 95% confidence intervals (CI) were calculated. For models 2 and 3, Adjusted odds ratio (AOR), 95% CIs, and Nagelkerke R² were calculated. Significance was set at *P* < 0.05. Data were analyzed using IBM SPSS statistical software (version 23 for Windows).

The DMFT index had a mean of 5.16 and SD of 5.04 in this sample (variance ≈ 25.4), a variance-to-mean ratio of approximately 4.9, indicating substantial overdispersion that supported the use of negative binomial rather than Poisson regression for this outcome. Given the 20 personality-trait–outcome associations tested across the four multivariable models, the Benjamini–Hochberg procedure was additionally applied to control the false discovery rate (FDR) at 5%.

## Results

**Fig. 1:**
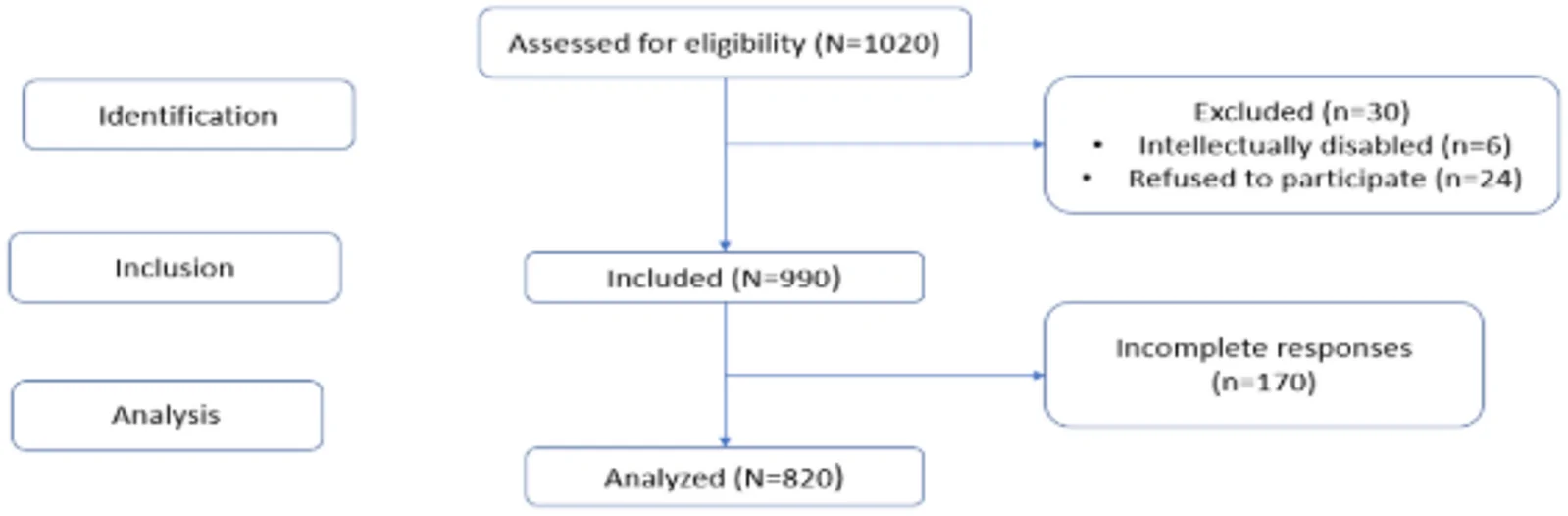
Flow chart of the flow of participants in the study

Of the 1,020 individuals assessed for eligibility, 30 were excluded (6 due to intellectual disability and 24 who declined to participate), leaving 990 eligible participants; a further 170 were excluded due to incomplete responses. A total of 820 participants were included in the study (response rate = 82.83%) **(**Fig. 1**)**. The mean ± SD age was 36.5 ± 13.0 years, 70.2% females, 60.0% with less than university level education, 45% lived in rural areas and 55.9% in urban areas.

Agreeableness had the highest score (mean ± SD = 11.53 ± 1.97) and openness had the lowest score (mean ± SD = 9.64 ± 2.27). The mean ± SD DMFT and sugar consumption scores were 5.16 ± 5.04 and 3.85 ± 2.06, respectively. About 59.1% brushed their teeth at least once daily and 60.6% visited a dental clinic last year **(**Table 1**)**.

**Table 1:** Description of the study participants (*N* = 820)

Age in years			Mean ± SD	36.5 ± 13.0
Gender			Female N (%)	576 (70.2%)
			Male N (%)	244 (29.8%)
Educational level			Below university N (%)	492 (60.0%)
			University or above N (%)	328 (40.0%)
Residence			Urban N (%)	458 (55.9%)
			Rural N (%)	362 (44.1%)
Personality traits score^1^	**Neuroticism**		Mean ± SD	9.72 ± 2.37
	**Extraversion**		Mean ± SD	9.71 ± 2.03
	**Openness**		Mean ± SD	9.64 ± 2.27
	**Agreeableness**		Mean ± SD	11.53 ± 1.97
	**Conscientiousness**		Mean ± SD	10.71 ± 2.04
Toothbrushing frequency^2^			Once or more daily N (%)	485 (59.1%)
			Less than once daily N (%)	335 (40.9%)
Dental visit last year^3^			Yes N (%)	497 (60.6%)
			No N (%)	323 (39.4%)
Sugar consumption score^4^			Mean ± SD	3.85 ± 2.06
Caries Experience (DMFT)	**Decayed (D)**		Mean ± SD Median (IQR)	2.02 ± 2.48 1.00 (0.00, 3.00)
	**Missing (M)**		Mean ± SD Median (IQR)	1.65 ± 3.17 0.00 (0.00, 2.00)
	**Filled (F)**		Mean ± SD Median (IQR)	1.48 ± 2.23 1.00 (0.00, 2.00)
	**DMFT index**		Mean ± SD Median (IQR)	5.16 ± 5.04 4.00 (2.00, 7.00)

*SD* Standard Deviation, *IQR* interquartile range

Scores were calculated as: 1. Big five personality traits (3) to (15) stronger expression of the corresponding personality trait, 4. (0) to (8) highest sugar consumption

2.Toothbrushing frequency (once or more daily vs. less than once daily) and 3. dental visit last year (Yes/No) were analyzed as binary variables

The Big Five personality traits showed no significant correlations with DMFT. Several traits were significantly correlated with sugar consumption: Openness (*r* = 0.13, *p* = < 0.001) and Conscientiousness (*r* = -0.09, *p* = 0.01). All Big Five traits except Conscientiousness were significantly associated with daily toothbrushing, indicating that personality influences oral hygiene practices. While only Openness was significantly associated with dental visits (*p* = 0.02 and 0.002, respectively), suggesting that individuals higher in openness are more likely to seek professional dental care **(**Table 2**)**.

**Table 2:** Association between DMFT and oral health related behaviors and the Big 5 constructs and confounders (*N* = 820)

Variables		Caries experience (DMFT)		Sugar consumption score		Daily Toothbrushing			Dental visit		
		Median (IQR)	*p* value	Mean ± SD	*p* value	Yes *N* (%)	No *N* (%)	*p* value	Yes *N* (%)	No *N* (%)	*p* value
Age in years	Mean ± SD ^d^	0.32	< 0.001*	− 0.14	< 0.001*	34.80 ± 12.23	38.92 ± 13.74	< 0.001*^b^	36.96 ± 12.78	35.75 ± 13.36	0.19^b^
Gender	Female N (%)	4.00 (2.00, 8.00)	0.01*^a^	3.84 ± 2.02	0.85 ^b^	373(64.80%)	203(35.20%)	< 0.001*^c^	361(62.70%)	215(37.30%)	0.06 ^c^
	Male N (%)	3.00 (1.00, 6.00)		3.87 ± 2.11		112(45.9%)	132(54.1)		136(55.70%)	108(44.30%)	
Educational level	University or above N (%)	2.00 (1.00, 5.00)	< 0.001*^a^	4.32 ± 2.01	< 0.001*^b^	288(87.8%)	40(12.2%)	< 0.001*^c^	222(67.70%)	106(32.30%)	0.003* ^c^
	Below university N (%)	5.00 (2.00, 9.00)		3.54 ± 2.03		197(40.00%)	295(60.00%)		275(55.90%)	217(44.10%)	
Residence	Urban N (%)	3.00 (1.00, 6.00)	< 0.001* ^a^	4.00 ± 2.03	0.02* ^b^	342(74.70%)	116(25.30%)	< 0.001*^c^	284(62.00%)	174(38.00%)	0.36 ^c^
	Rural N (%)	5.00 (2.00, 9.00)		3.66 ± 2.07		143(39.5%)	219(60.50%)		213(58.80%)	149(41.20%)	
Neuroticism	Mean ± SD ^d^	-0.03	0.41	0.04	0.31	9.53 ± 2.29	10.00 ± 2.45	0.01*^b^	9.74 ± 2.38	9.70 ± 2.35	0.79^b^
Extraversion	Mean ± SD ^d^	0.05	0.14	-0.003	0.93	9.59 ± 2.04	9.89 ± 2.01	0.04*^b^	9.69 ± 2.05	9.74 ± 2.01	0.71^b^
Openness	Mean ± SD ^d^	-0.03	0.38	0.13	< 0.001*	9.85 ± 2.28	9.35 ± 2.23	0.002*^b^	9.80 ± 2.29	9.41 ± 2.23	0.02* ^b^
Agreeableness	Mean ± SD ^d^	-0.04	0.26	0.01	0.78	11.72 ± 2.02	11.25 ± 1.86	0.001*^b^	11.57 ± 2.03	11.46 ± 1.87	0.46^b^
Conscientiousness	Mean ± SD ^d^	0.003	0.94	-0.09	0.01*	10.79 ± 2.05	10.60 ± 2.03	0.19 ^b^	10.79 ± 2.07	10.58 ± 2.00	0.15^b^

*SD* Standard Deviation, *IQR* Interquartile range

^a^Mann Whitney U test. ^b^Independent t test. ^c^ Chi-square test, d: Spearman correlation was used for DMFT and Pearson correlation for sugar consumption scores; values represent correlation coefficients rather than mean ± SD

*Statistically significant at *p* < 0.0

In multivariable regression (Table 3), agreeableness was significantly associated with lower DMFT scores (B = -0.04, 95% CI = -0.09, 0.00, *p* = 0.04); no other trait reached significance (Table 3). The model explained 21% of the variance in DMFT (Cox & Snell pseudo- R² = 0.21; McFadden’s pseudo-R² = 0.04).

**Table 3:** Regression analysis for the association between Big five traits and DMFT, sugar consumption score, frequent toothbrushing and dental visits last year (*N* = 820)

Explanatory variables		DMFT				Sugar consumption score		Frequent toothbrushing		Dental visits last year	
		B (95% CI)	IRR (exp[B])		*p* value	B (95% CI)	*p* value	AOR (95% CI)	*p* value	AOR (95% CI)	*p* value
Age		0.02 (0.02, 0.03)	1.02 (1.02, 1.03)		< 0.001*	-0.01 (-0.03, -0.003)	0.01*	0.99 (0.98, 1.01)	0.27	1.02 (1.00, 1.03)	0.01*
Gender	Female	0.36 (0.18, 0.54)	1.43 (1.20, 1.72)		< 0.001*	-0.05 (-0.37, 0.27)	0.77	3.01 (2.05, 4.41)	< 0.001*	1.38 (0.99, 1.91)	0.06
	Male (ref category)	0	0			0		1		1	
Educational level	Below university	0.31 (0.14, 0.49)	1.36 (1.15, 1.63)		< 0.001*	-0.65 (-0.97, -0.33)	< 0.001*	0.19 (0.13, 0.27)	< 0.001*	0.65 (0.46, 0.91)	0.01*
	University & higher (ref category)	0	0			0		1		1	
Residence	Urban	-0.27 (-0.43, -0.11)	0.76 (0.65, 0.90)		0.001*	0.08 (-0.23, 0.39)	0.63	3.17 (2.26, 4.45)	< 0.001*	1.03 (0.76, 1.40)	0.85
	Rural (ref category)	0	0			0		1		1	
Neuroticism		-0.02 (-0.06, 0.01)	0.98 (0.94, 1.01)		0.16	0.03 (− 0.03, 0.09)	0.35	0.90 (0.83, 0.96)	0.003*	1.01 (0.95, 1.08)	0.77
Extraversion		0.01 (-0.03, 0.05)	1.01 (0.97, 1.05)		0.65	0.03 (− 0.04, 0.10)	0.43	1.01 (0.93, 1.09)	0.90	0.98 (0.91, 1.05)	0.58
Openness		0.02 (-0.02, 0.06)	1.02 (0.98, 1.06)		0.35	0.12 (0.06, 0.19)	< 0.001*	1.06 (0.98, 1.14)	0.16	1.08 (1.01, 1.15)	0.03*
Agreeableness		-0.04 (-0.09, 0.00)	0.96 (0.91, 1.00)		0.04*	0.01 (− 0.06, 0.09)	0.75	1.07 (0.98, 1.17)	0.14	0.98 (0.90, 1.06)	0.54
Conscientiousness		-0.01 (-0.05, 0.03)	0.99 (0.95, 1.03)		0.70	−0.15 (− 0.22, − 0.08)	< 0.001*	0.99 (0.91, 1.08)	0.77	1.03 (0.95, 1.11)	0.47
R²/pseudo-R² ¹				**0.21**		**0.06**		**0.35**		**0.03**	

B regression coefficient

AOR Adjusted odds ratio; IRR Incidence rate ratio (exp[B]), reported for the DMFT model

¹Model fit statistics differ by model type and are not directly comparable across columns: Adjusted R² is reported for the linear (sugar consumption) model; Cox & Snell pseudo-R² is reported for the negative binomial (DMFT) model (the more conservative McFadden’s pseudo-R² for the same model is 0.04); Nagelkerke pseudo-R² is reported for the two logistic (toothbrushing, dental visit) models

*Statistically significant at *p* < 0.05

Models were adjusted for age, gender, educational level and residence

Agreeableness was not significantly correlated with DMFT in the unadjusted bivariate analysis (Table 2) but became significant after adjustment for age, gender, educational level, and residence, a pattern consistent with confounding or suppression by these socio-demographic variables.

Openness was associated with significantly higher sugar consumption score (B = 0.12, 95% CI = 0.06, 0.19, *p* < 0.001), while Conscientiousness was associated with significantly lower scores (B = -0.15, 95% CI = -0.22, 0.08, *p* < 0.001); no other trait was significant (Table 3). The model explained 6% of the variance in sugar consumption score (Adjusted R² = 0.06).

Only neuroticism was associated with significantly lower odds of daily toothbrushing (AOR = 0.90, 95% CI = 0.83, 0.96, *p* = 0.003); the remaining traits were not significant (Table 3). The model accounted for 35% of the variance in frequent toothbrushing (Nagelkerke R² = 0.35).

Only openness was associated with significantly higher odds of dental visits last year (AOR = 1.08, 95% CI = 1.01, 1.15, *p* = 0.03); no other trait reached significance (Table 3). The model explained 3% of the variance ² (Nagelkerke R² = 0.03).

After applying the Benjamini–Hochberg correction for the 20 personality-trait–outcome tests across the four multivariable models, only the associations of conscientiousness and openness with sugar consumption and of neuroticism with toothbrushing frequency remained significant; the associations of agreeableness with DMFT and of openness with dental visits did not survive correction for multiple testing and should be interpreted with caution.

## Discussion

The study findings indicated that, among underserved Egyptian adults, various personality traits were associated with caries experience and oral health behaviors. Agreeableness was associated with lower caries experience, openness and less conscientiousness were associated with higher sugar consumption, neuroticism was associated with lower odds of frequent toothbrushing, and openness was linked to greater odds of dental visits last year. Thus, the null hypothesis was rejected.

This study has some limitations. First, cross-sectional design cannot prove causality, only associations. Longitudinal studies are needed to assess the temporal associations between personality traits, and oral health outcomes [32]. Second, most participants were females, as men were at work during the study visits. which may have led to underrepresentation of Egyptian males whose personality traits differ from females [6]. Third, social desirability bias may have affected self-reported behavior, potentially leading to over reporting of favorable oral health behavior and socially desirable personality traits [33]. Fourth, Although the BFI-2-XS was translated by a bilingual translator and back-translated by an independent bilingual translator, its Arabic version has not been formally psychometrically validated, which may have attenuated some trait-outcome associations [34]. Fifth, convenience sampling may have introduced selection bias, for example, by preferentially including individuals already engaged with outreach programs or by excluding individuals who were unable to complete the self-administered questionnaire because of the required literacy requirement. Therefore, the findings should be generalized cautiously to underserved populations with similar recruitment characteristics rather than to underserved populations in general [33]. Nevertheless, the sample included participants from both rural and urban areas, including Bedouin communities, which may enhance the applicability of the findings to underserved communities with similar characteristics.

Nevertheless, the study has several points of strength. First, community-based recruitment allowed the inclusion of underserved populations in their own settings rather than in clinic-based environments, which can enhance representativeness and external validity [33]. Second, the study provides evidence on the association between personality traits, dental caries experience, and oral health-related behaviors among underserved adults residing in a developing country, contributing to the literature on psychosocial influences on oral health, which mostly comes from developed countries [21].

Agreeableness was associated with lower dental caries experience. One possible explanation is that agreeable individuals tend to have lower levels of chronic stress and stronger social support that facilitate a favorable health status [35]. This is in line with a study showing a non-significant correlation between higher agreeableness and lower dental caries experience among Mexican dental patients [4].

Neuroticism was associated with lower odds of frequent toothbrushing which could be explained by the tendency of neurotic individuals to suffer from anxiety and emotional distress, potentially reducing their adherence to healthy practices, including toothbrushing [35, 36]. This is consistent with a study showing that neuroticism was significantly associated with reduced toothbrushing among middle-class community-dwelling older adult Americans [37].

Openness, on the other hand, was linked to a greater odd of dental visits. This could be attributed to open individuals’ proactivity [38], which would motivate them to use healthcare services [14]. At the same time, openness is often associated with sociability, social eating and experimentation [17], which may result in more sugar consumption observed in this study. Thus, openness can simultaneously promote healthcare seeking while increasing the exposure to dietary risk factors. This agrees with a longitudinal study showing that American adults with high levels of openness non-significantly tended to use more healthcare services [15].

Meanwhile, conscientiousness was related to lower levels of sugar consumption. One possible explanation is that conscientious individuals typically show higher resilience, self-discipline and regulation of indulgent behaviors [35, 39], which support healthier dietary choices resulting in reduced intake of sugary foods and beverages [40]. This agrees with a study showing significant association between conscientiousness and better oral health behavior among Jordanian dental students [41].

Extraversion did not show significant associations with either oral diseases or oral health-related behaviors. One possible explanation is that opposing pathways of extraversion can offset one another. Extroverted individuals may benefit from stronger social engagement, supporting good oral health [35, 42], yet they may also engage more frequently in unhealthy behaviors [43], including social eating [44], resulting in conflicting net effects on oral health outcomes or behaviors. This is consistent with a study showing that extroversion was non-significantly associated with toothbrushing among American middle-class community-dwelling older adult men [37], or sugar intake among American millennials [45].

Overall, the associations between personality traits and dental caries as well as oral health behaviors were weak, despite some statistically significant relationships. This is likely because psychosocial influences interact within a multifactorial framework influencing oral health, which includes individual, familial, and community-level determinants [46]. The effects of personality traits may be overshadowed by stronger risk factors such as mother’s educational level, socioeconomic status, dental insurance, and dental care system characteristics [47], which characterize underserved communities.

The null finding for extraversion may partly reflect measurement limitations, as this trait was assessed with only three items in the BFI-2-XS, limiting score range and reliability; the predominantly female composition of the sample, which narrowed the observed range of extraversion scores; and cultural context, since the behavioral expression of sociability and its relationship to dental care-seeking may differ from the largely Western samples in which most cited extraversion findings were generated.

Although several of these associations were statistically significant, their absolute effect sizes were modest: openness and conscientiousness shifted sugar consumption by well under one point on the 8-point scale per unit of trait score, and agreeableness shifted DMFT only marginally. Personality traits are therefore unlikely to be useful as standalone screening tools but may complement core behavioral and socioeconomic interventions.

More broadly, model fit statistics across the four regression models were consistently modest, though they are not directly comparable across models given differing metrics: Adjusted R² = 0.06 for the sugar-consumption model; Cox & Snell pseudo-R² = 0.21 (McFadden’s pseudo-R² = 0.04) for the negative binomial DMFT model; and Nagelkerke pseudo-R² = 0.03–0.35 for the toothbrushing and dental-visit logistic models. The higher end of this range was driven mainly by the socio-demographic confounders (age, gender, educational level, residence) rather than by the personality traits themselves. Statistical significance in a sample of this size should therefore not be read as evidence that personality traits are strong or clinically actionable predictors of caries experience or oral health behaviors; these associations are better interpreted as modest correlates that may inform, but should not by themselves drive, targeting of preventive interventions.

Within an ecological, social-determinants-of-health framework, personality operates as one individual-level factor nested within family, community, and health-system-level determinants. Notably, educational level was a more consistent predictor than any single personality trait across all four regression models in this study (Table 3), underscoring the likely greater population-level impact of structural determinants and supporting multi-level prevention strategies that combine structural and individual-level approaches.

Prevention of oral diseases is specifically important in underserved areas where access to dental care is limited, and the cost of treatment is often unaffordable. Community-embedded oral health education programs should address the relationship between individual characteristics and oral health behaviors. Interventions aimed at reducing emotional distress may be beneficial, including the provision of mental health support, as positive mental well-being is linked to improved oral health behaviors and reduced risk of stress-related oral conditions [36]. Building resilience and effective coping strategies among individuals experiencing emotional distress through structured training programs may further support positive oral health behaviors. Future longitudinal research should incorporate additional oral health determinants, such as cultural dietary norms and household economic constraints, to better explain variations in oral health status and oral health behaviors beyond personality traits.

## Conclusion

Certain personality traits were significantly associated with dental caries experience and oral health behaviors among Egyptians in underserved areas. Incorporating psychological characteristics into community-based oral health promotion strategies may enhance preventive behaviors and reduce oral health disparities.

## Data Availability

The datasets generated and/or analyzed during the current study are available from the corresponding author on reasonable request.
